# Personalizing the safe, appropriate and effective concentration(s) of ozone for a non-diabetic individual and four type II diabetic patients in autohemotherapy through blood hemoglobin analysis

**DOI:** 10.1186/s12967-019-1973-5

**Published:** 2019-07-16

**Authors:** Fouad Mehraban, Arefeh Seyedarabi, Shahin Ahmadian, Vahid Mirzaaghaei, Ali Akbar Moosavi-Movahedi

**Affiliations:** 1 Institute of Biochemistry and Biophysics, University of Tehran, Tehran, Iran; 2 Gardina Corporation, Tehran, Iran

**Keywords:** Autohemotherapy, Antioxidants, Diabetes, Personalized ozone therapy, Whole blood hemoglobin

## Abstract

**Background:**

Diabetes is a chronic disease associated with many problems and high costs. In recent decades, a lot of research has been carried out in order to improve methods of treatment of diabetic patients. One of the currently used complementary therapies for diabetes is ozone therapy or autohemotherapy. The beneficial effects of ozone has been proven in many diseases such as diabetes, but the critical issue is the determination of the safe and effective concentration of ozone reacting with blood and in particular hemoglobin.

**Methods:**

A number of spectroscopic techniques including intrinsic fluorescence, circular dichroism and UV–VIS spectroscopies were used as well as SDS-PAGE, Native-PAGE and dynamic light scattering to analyze the effect of ozonation on hemoglobin of a non-diabetic individual and four diabetic patients in order to find the appropriate concentration(s) of ozone for personalized autohemotherapy.

**Results:**

In this study, we determined the personalized concentration(s) for a safe and effective ozonation of a non-diabetic individual and four diabetic type II patients, based on blood hemoglobin analysis.

**Conclusions:**

A number of techniques were used to determine the personalized ozone concentration(s) for a safe and effective autohemotherapy based on blood hemoglobin analysis. SDS-PAGE and dynamic light scattering were identified as the two main techniques needed for personalizing the ozone concentration(s) for each individual as otherwise hemoglobin in blood can oligomerise and cause serious damage if the inappropriate ozone concentration is used.

## Background

Diabetes is a metabolic disease characterized by impaired carbohydrate metabolism, inappropriate insulin production or consumption, leading to glycosuria and hyperglycemia [1]. The global prevalence of diabetes is increasing substantially. In total, since 2011, 366 million people have been diagnosed with diabetes, with type II diabetes accounting for almost 90% of all cases [2]. Type II diabetes together with type I diabetes or insulin-dependent diabetes are amongst the toughest conditions in terms of their social and economic pressures and the suffering caused. In developed countries, the number of diabetic patients is steadily rising and the rate of disability and mortality are also increasingly seen [3, 4]. In patients with type II diabetes, which is characterized by the inability of tissues to detect insulin-sensitivity, the process of gluconeogenesis is increased in the liver where glucose uptake and its conversion in insulin-sensitive tissues are severely impaired. To eliminate these disorders, the release of insulin by beta cells increases and leads to type II diabetes, which is characterized by high levels of plasma glucose and the presence of hyperinsulinemia [5].

There is a lot of clinical evidence that has shown the increase in the production of reactive oxygen species (ROS) in both types of diabetes [6]. There is also a direct relationship between the presence of oxidative stress and the defective uptake of glucose. Antioxidant depletion associated with a reduction in the uptake of glucose in patients with type II diabetes has been reported. The imbalance in the level of active oxygen species and antioxidants plays an important role in insulin resistance, and other studies have shown that there is a close relationship between oxidative stress and insulin sensitivity. Therefore, it seems that increasing the antioxidant capacity can overcome insulin resistance [5].

Ozone is one of the most reactive gases and the third most potent oxidant after fluorine and persulfate [7] and ozone therapy has been recorded amongst the bio-oxidative therapies, which has been used and studied for its use as a disinfectant and for the treatment of different diseases including diabetes [8]. In the phenomenon of adaptation to chronic oxidative stress [8], which is also evaluated as oxidative pre-conditioning [9] or as hormosis [10, 11], it has been determined that repetitive and calculated treatment methods such as ozone therapy with the appropriate concentration and manner [12] can have a stimulated effect at concentrations lower than the inhibitory and toxic amounts [11] triggering the synthesis of oxidative stress proteins such as HO-1 [13]. Indeed, the therapeutic efficacy of ozone therapy may also be partly due to the controlled and moderate oxidative stress produced by the reaction of ozone with several biological components [11].

It has been reported that ozone therapy, through the mentioned mechanism, returns glucose levels to normal and subsequently returns the concentrations of organic peroxides to its original state and reduces hyperglycemia in patients with type II diabetes and exerts anti-diabetic effects [5]. Ozone can maintain the content of glycogen, reduce the formation of lactate and uric acid, and also stimulate or maintain endogenous antioxidant systems and block the xanthine-oxidase pathway, which produces ROS, through the process of oxidative pre-conditioning and controlling the damage caused from oxidative stress by CCl_4_ [14–16]. Endogenous oxidative stress has a long half-life and it is different from temporary, precise and regulated oxidative stress caused by exposure of ex vivo blood of patients to oxygen and ozone gas mixture. Ozone therapy which can create this type of gentle, precise and temporary oxidative stress may reduce chronic oxidative stress and possibly the complications of diabetes [1]. Indeed, according to recent studies on the biological activity of ozone, it has been proven that low and carefully adjusted ozone concentrations unexpectedly induce an adaptive response that can reduce the endogenous oxidative stress [8, 17, 18].

One of the most common and effective methods of ozone therapy for patients is autohemotherapy (O_3_-AHT), which involves the exposure of a certain amount of blood from the patient, along with sodium citrate as an anticoagulant, with an equal volume of oxygen (95%) and ozone (5%) gas mixtures, at a specific concentration per ml of blood [12, 19].

Ozone in the aqueous environment, which is able to quickly dissolve, gives its energy to hematic components [20], such as hemoglobin (Hb), the most heme-containing protein in the blood circulation. Therefore, the effects of ozone on Hb is widely investigated [21].

The body can deal with oxidative stress via the production of endogenous or exogenous antioxidants. Antioxidants play their role by neutralizing excess free radicals and protecting cells against toxic effects and helping prevent the prevalence of disease [22]. Total antioxidant capacity (TAC) values vary in non-diabetic individuals and diabetic patients [23]. Plasma TAC measurements are considered as an important step in explaining the relationship between antioxidant status and other diseases [24].

In this study, we investigated for the first time, the effect of various concentrations of ozone on human Hb in the whole blood of a non-diabetic individual and four diabetic patients (with type II diabetes), in order to place emphasis on a personalized method for the identification of a safe, appropriate and effective concentration(s) of ozone for treatment of diabetic patients in O_3_-AHT. This study involved the use of spectroscopic studies such as the intrinsic fluorescence, circular dichroism (CD) and UV–VIS spectroscopies, in order to examine the changes in the secondary and tertiary structure of Hb and its heme group and aromatic residues, as well as Native-PAGE and in particular SDS-PAGE, in the presence and absence of dithiothreitol (DTT) (as a reducing agent), and dynamic light scattering (DLS) to analyze the oligomerization and polydispersity of Hb upon exposure to varying concentrations of ozone.

## Methods

### Ozone generation

Ozone was produced from medical-grade oxygen by an electrical corona arc discharge by an O_3_ generator (Gardina, Iran), which controls the amount of gas flow rate and ozone concentration using a photometry, periodically checked by the iodometric titration in accordance with the rules established by the international ozone association [25]. Blood ozonation should only be done with medical oxygen and not the filtered air which contains 78% nitrogen resulting in the unwanted formation of nitrogen oxides. Single-use silicon treated polypropylene syringes (ozone resistant) were used throughout the reaction time to ensure the stability of the ozone concentration and to prevent its leakage or contamination.

### Collection, ozonation and purification of Hb

Hb in whole blood samples of the non-diabetic individual and four diabetic patients were ozonated with different concentrations of ozone at 15, 35 and 55 µg/ml, purified and then dialyzed by the method used in our previous study [26].

### Gas delivery and whole blood sample treatments

In our experiments, a single concentration of ozone gas mixture (concentration per volume, µg/ml) composed of oxygen (96–99%) and ozone (1–4%) with flow rate of 0.8 lit/min was used. The final pressure of the gas was maintained at a normal atmospheric pressure [27]. In our previous study, we reported the amount of ozone used in terms of ‘ppm’ and ‘dose’ [26]. However, in this study we are using ‘µg/ml’ and ‘concentration’, in place of those terms, as a more accurate measurement of ozone gas. The various ozone concentrations in major O_3_-AHT used for treatment of diabetic patients are 15, 35 and 50 µg/ml, which are also effective in peripheral arterial disease [28], with the best time duration for the effective mixing with blood being 5 min [19]. This time duration is needed for blood (which is viscous) to achieve complete and homogeneous equilibrium with ozone gas [29].

In this study, whole blood samples were exposed to ozone gas at 15, 35 and 55 µg/ml of ozone for 5 min at a 1:1 volumetric ratio, followed by Hb purification [27]. 55 µg/ml was chosen instead of the reported 50 µg/ml in order to keep consistency in the increased ozone concentration levels used in this study. The control blood samples were not exposed to ozone.

### Fluorescence spectroscopy

The fluorescence spectra were examined with the same apparatus and method as described in our previous study [26]. Emission spectra were measured between 300 and 400 nm. The excitation wavelength was at 280 nm.

### CD measurements

Measurements of the CD spectra were carried out using an AVIV 215 spectropolarimeter (Aviv Associates, Lakewood, NJ, USA). All CD measurements were carried out as in our previous study [26]. The results were plotted as ellipticity (in deg. cm^2^ dmol^−1^) versus wavelength in nanometers.

### UV–VIS absorption spectroscopy

The same parameters as in our previous study were used to measure the UV–VIS spectra [26]. The absorbance scan spectra were measured using a UV–visible spectrophotometer (Varian, Carry 100 Bio, Australia) at an absorbance of 280 nm and reported in the range of 200–700 nm.

### SDS-PAGE and Native-PAGE

In SDS-PAGE and Native-PAGE analyses, all conditions were the same as in our previous study and 18% gels were used [26]. Molecular weights of bands in the Hb samples were estimated by comparison with a protein marker (SMOBiO, Taiwan).

### Dynamic light scattering

DLS measurements of Hb from the non-diabetic individual and that of the four diabetic patients were obtained using the same method as reported in our previous study [26]. The refractive index of the material (protein) and the absorption were set to 1.59 and 0.01, respectively. The following parameters were used: the dispersant viscosity was 0.8872 cP and the refractive index was 1.330.

### Measurement of plasma TAC

The TAC values derived from blood plasma of the non-diabetic individual and that of the four diabetic patients prior to ozone therapy were measured using the ZellBio GmbH kit (product of Germany) and expressed in mM [30].

### Statistical analysis

The TAC results were averages of three measurements from blood plasma of each individual and reported as mean ± SD by the Friedman test (a non-parametric test) and presented using the SPSS software, version 18.

## Results

### Non-diabetic and type II diabetic patients

Table 1 provides information about the non-diabetic individual and the four diabetic patients (including duration of disease), the TAC measurements from each individual’s blood plasma (measured three times and averaged) and the purified Hb concentration of each sample after ozonation based on the absorbance reading at 280 nm (which is dependent on the presence or exposure of aromatic ring containing residues, in particular tryptophan and tyrosine). In this study, Hb from whole blood of a non-diabetic individual and four patients diagnosed with type II diabetes were analysed, in order to determine the safe and effective concentration(s) of ozone to be used for each individual in O_3_-AHT.

**Table 1:** General information for the non-diabetic individual A and the four diabetic patients B–E

Samples	Age	Gender	Hb concentration (mg/ml) based on absorbance reading at 280 nm post ozonation and dialysis	TAC (mM)	Duration of disease (medication taken)
Individual A	46	Male	Non-O_3_: 100.70 15 μg/ml O_3_: 136.48 35 μg/ml O_3_: 110.01 55 μg/ml O_3_: 111.48	0.274 ± 0.009	–
Patient B	56	Female	Non-O_3_: 86.63 15 μg/ml O_3_: 79.61 35 μg/ml O_3_: 103.14 55 μg/ml O_3_: 75.38	0.207 ± 0.006	18 years (Metformin)
Patient C	66	Male	Non-O_3_: 66.12 15 μg/ml O_3_: 94.41 35 μg/ml O_3_: 80.59 55 μg/ml O_3_: 70.15	0.341 ± 0.015	20 years (Insulin)
Patient D	60	Male	Non-O_3_: 48.41 15 μg/ml O_3_: 63.92 35 μg/ml O_3_: 74.52 55 μg/ml O_3_: 86.88	0.370 ± 0.013	1 year (Metformin)
Patient E	41	Female	Non-O_3_: 62.80 15 μg/ml O_3_: 59.38 35 μg/ml O_3_: 57.40 55 μg/ml O_3_: 55.65	0.271 ± 0.029	5 years (Insulin)

Looking at the results of Hb concentrations based on absorbance reading at 280 nm after ozonation, there seemed to be an indication that the Hb concentrations varied amongst the samples. This could be related to structural changes caused by ozonation such that the aromatic residues, which have absorbance at 280 nm, were exposed differently for detection.

In the non-diabetic individual A, the concentration of Hb in each of the three ozone treated concentrations compared to the non-ozonated Hb sample from the same individual had increased. The maximum Hb concentration was measured for Hb ozonated with 15 µg/ml ozone. In patient B, the highest concentration of Hb was measured for Hb ozonated with 35 µg/ml ozone. In patient C, the highest concentration of Hb was in the presence of ozone at 15 µg/ml. In patient D, the highest concentration of Hb was in the presence of ozone at 55 μg/ml and in patient E the highest concentration was for the non-ozonated Hb sample. These observations will be better explained with other techniques used in this study.

### Ozonation of Hb

**Fig. 1:**
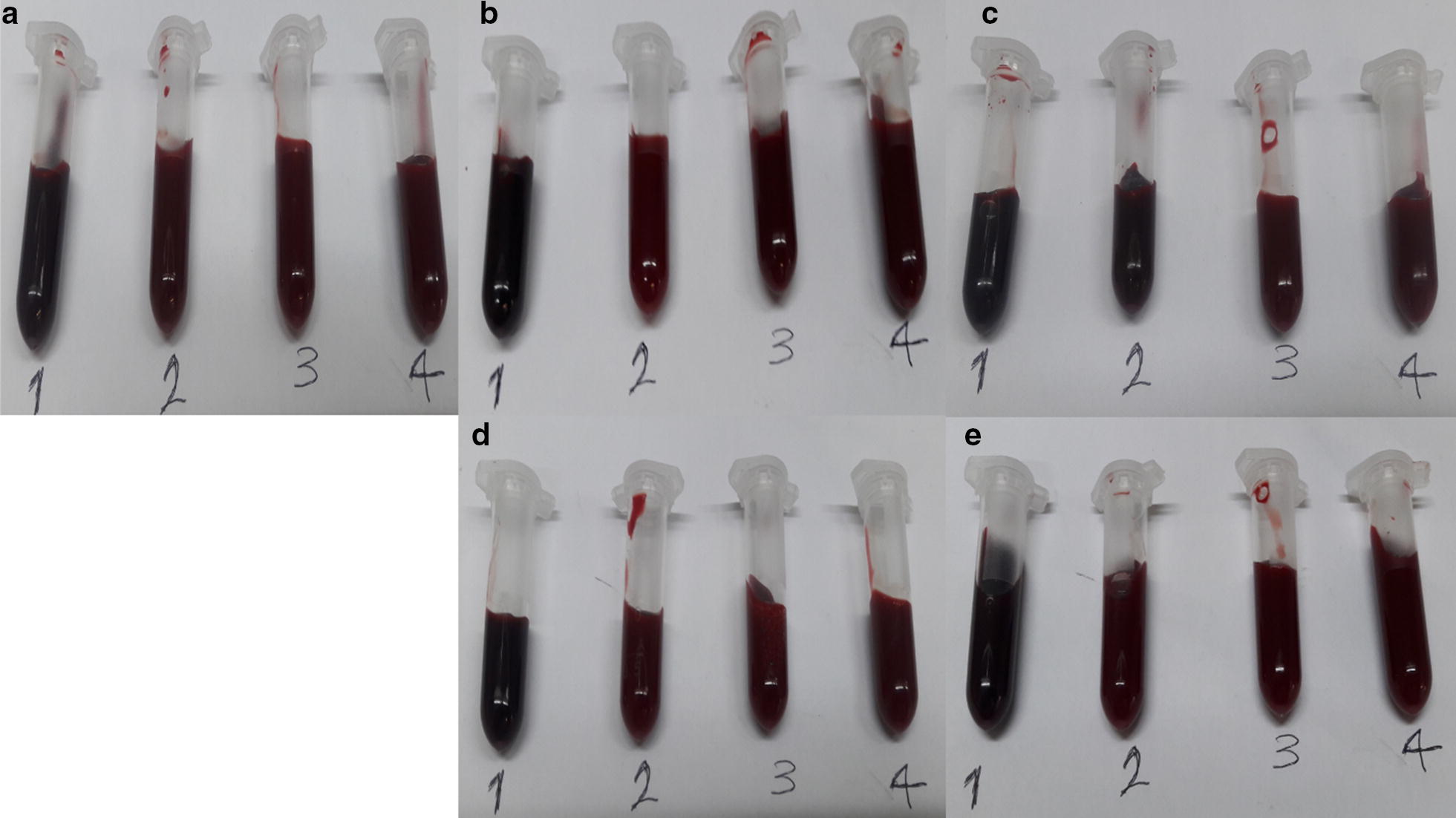
Visual colour representation of oxy-Hb from five human blood samples. **a** Hb of the non-diabetic individual A. **b**–**e** Hb of the diabetic patients B to E, respectively. In all cases, sample number 1 refers to non-ozonated Hb, while samples number 2, 3 and 4 are related to ozone treated Hb with 15, 35 and 55 μg/ml of ozone, respectively, for 5 min

In this study, whole blood Hb was purified from a non-diabetic individual and four diabetic patients after exposure to various concentrations of ozone (15, 35 and 55 µg/ml) for 5 min. In all cases, a slight change in blood colour from dark red in control samples to slightly bright red in ozonated samples at different concentrations were observed (Fig. 1). This indicated that ozonation affected the colour of the whole blood Hb slightly, but nowhere close to that observed in our previous study where the absence of antioxidants and the direct continuous bubbling of ozone in purified Hb samples resulted in eye catching colour changes [26], as opposed to whole blood Hb samples in this study, which are in the presence of antioxidants (with TAC values given in Table 1) and exposed to ozone gas in a 1:1 volumetric ratio in a syringe.

### Fluorescence analysis

Fluorescence is used to evaluate changes in the tertiary structure of proteins such as Hb [31], which are characterized by their own intrinsic inherent fluorophores including that of tyrosines, phenylalanines and predominantly tryptophans. There is a β-Trp37 located in the α1β2 interface which seems to play a major role in the intrinsic fluorescence of Hb [32]. In the non-diabetic individual A, no change in intensity of fluorescence peak was observed for Hb exposed to different concentrations of ozone compared to its control non-ozonated Hb sample, consistent with our previous study [26].

**Fig. 2:**
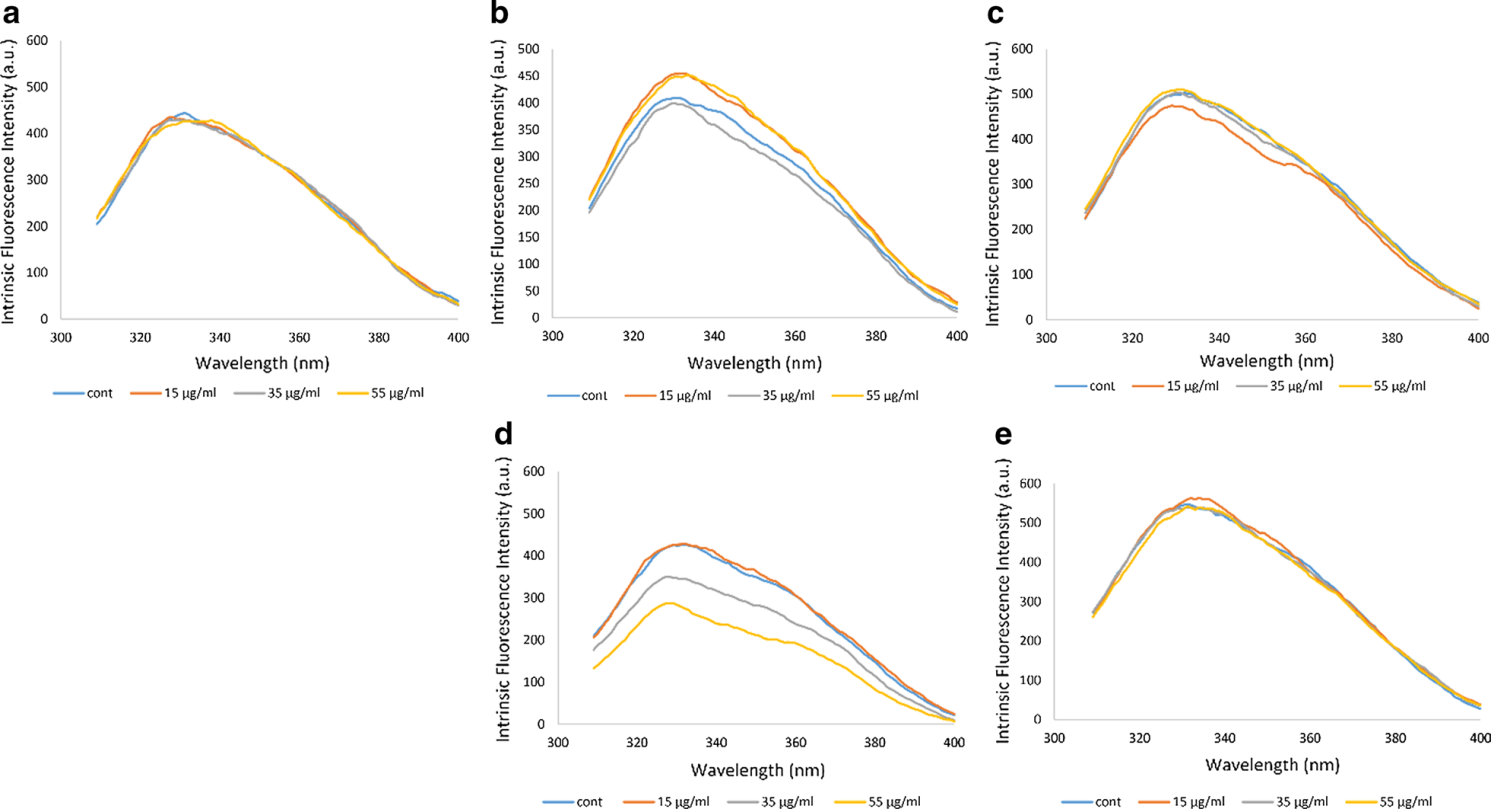
Evaluating the intrinsic fluorescence spectral changes of oxy-Hb from five human blood samples. The fluorescence spectra were measured after excitation at 280 nm. **a** Fluorescence spectra of oxy-Hb for the non-diabetic individual A. **b**–**e** Fluorescence spectra of oxy-Hb for the diabetic patients B to E, respectively

In the diabetic patient B, the ozone concentration of 35 μg/ml had reduced the Hb peak intensity while ozone concentrations of 15 μg/ml and 55 μg/ml had increased the intensity of the Hb peak. In the diabetic patient C, the ozone concentration of 15 μg/ml had reduced the Hb peak intensity slightly. In the diabetic patient D, ozone concentrations of 55 μg/ml followed by 35 μg/ml had reduced the peak intensities, while ozone concentration of 15 μg/ml was able to raise the peak intensity close to that seen for the non-ozonated Hb sample. In the diabetic patient E, there was no major change in peak intensity for the varying ozone concentrations. What is interesting is the direct relationship between the Hb concentrations as listed in Table 1 and the intrinsic fluorescent peaks seen in Fig. 2. For example, the trend for the highest to lowest concentrations (based on absorbance readings at 280 nm) of Hb for patient B is related to Hb ozonated at 35 μg/ml, followed by non-ozonated, then 15 μg/ml and finally 55 μg/ml ozone. This trend is also consistent in Fig. 2b, where the lowest peak with the highest intrinsically exposed Hb structure is related to Hb treated with 35 μg/ml ozone. This is much more clearly apparent for patient D at 55 μg/ml. The reason for this could be that ozone concentrations of 35 and 55 μg/ml used for Hb samples of patients B and D, respectively, caused a change and polarization in the surrounding environment of the fluorophores and that of β-Trp37 in Hb, in particular, resulting in lower fluorescence peak intensity and a more open structure compared to the non-ozonated and other ozone treated Hb samples for these patients.

### CD measurements

#### Far-UV CD

**Fig. 3:**
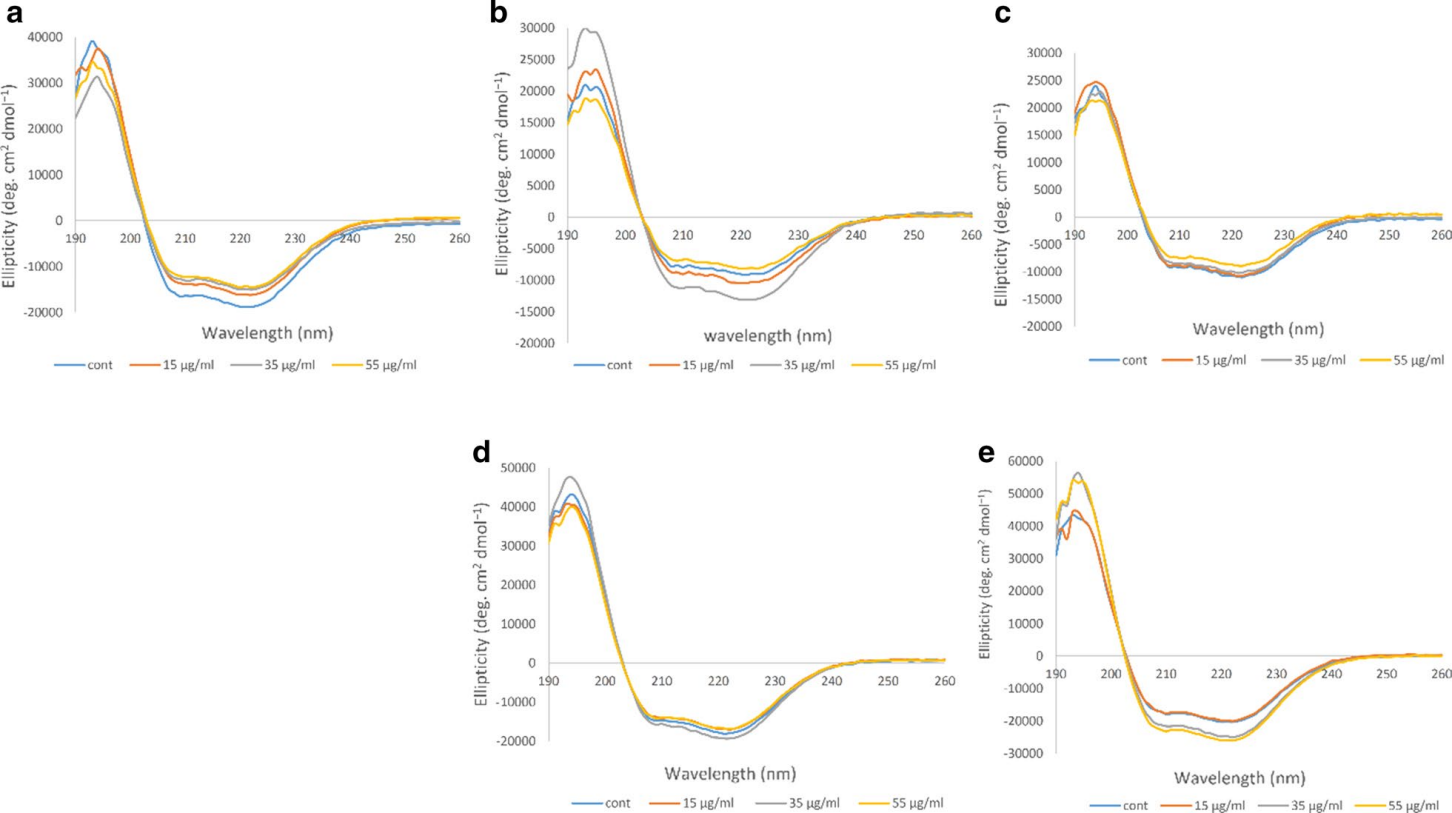
Far-UV CD spectral changes of ozonated whole blood oxy-Hb from five human samples. **a** Far-UV spectra of oxy-Hb for the non-diabetic individual A. **b**–**e** Far-UV spectra of oxy-Hb for the diabetic patients B to E, respectively

CD measurements are used to study changes in the structure of proteins [31]. In far-UV CD the alpha helical structure of a protein is detected through two highly negative peaks at 209 nm and 222 nm, which are related to the π–π* transition in α-helix and n-π* transition in both α-helix and random coil conformations, respectively [33, 34]. As you can see in Fig. 3, in the non-diabetic individual A, we have the most negative peaks with highest alpha helix content for the non-ozonated Hb sample, which decreases in intensity when treated with 15 μg/ml ozone. In the diabetic patient B, minor changes were observed for Hb samples treated with 15 μg/ml and 55 μg/ml ozone compared to the non-ozonated Hb sample, while ozone concentration of 35 μg/ml resulted in pronounced increase in peak intensities and an increase in the alpha helix content. In the diabetic patient C, the intensity of both peaks was not significantly different between the non-ozonated and ozone treated Hb samples. In the diabetic patient D, changes were also negligible between the non-ozonated and ozone treated Hb samples. As for the diabetic patient E, ozone concentrations of 55 μg/ml followed by 35 μg/ml showed a noticeable increase in peak intensities indicative of increased alpha helix content.

#### Near-UV CD

**Fig. 4:**
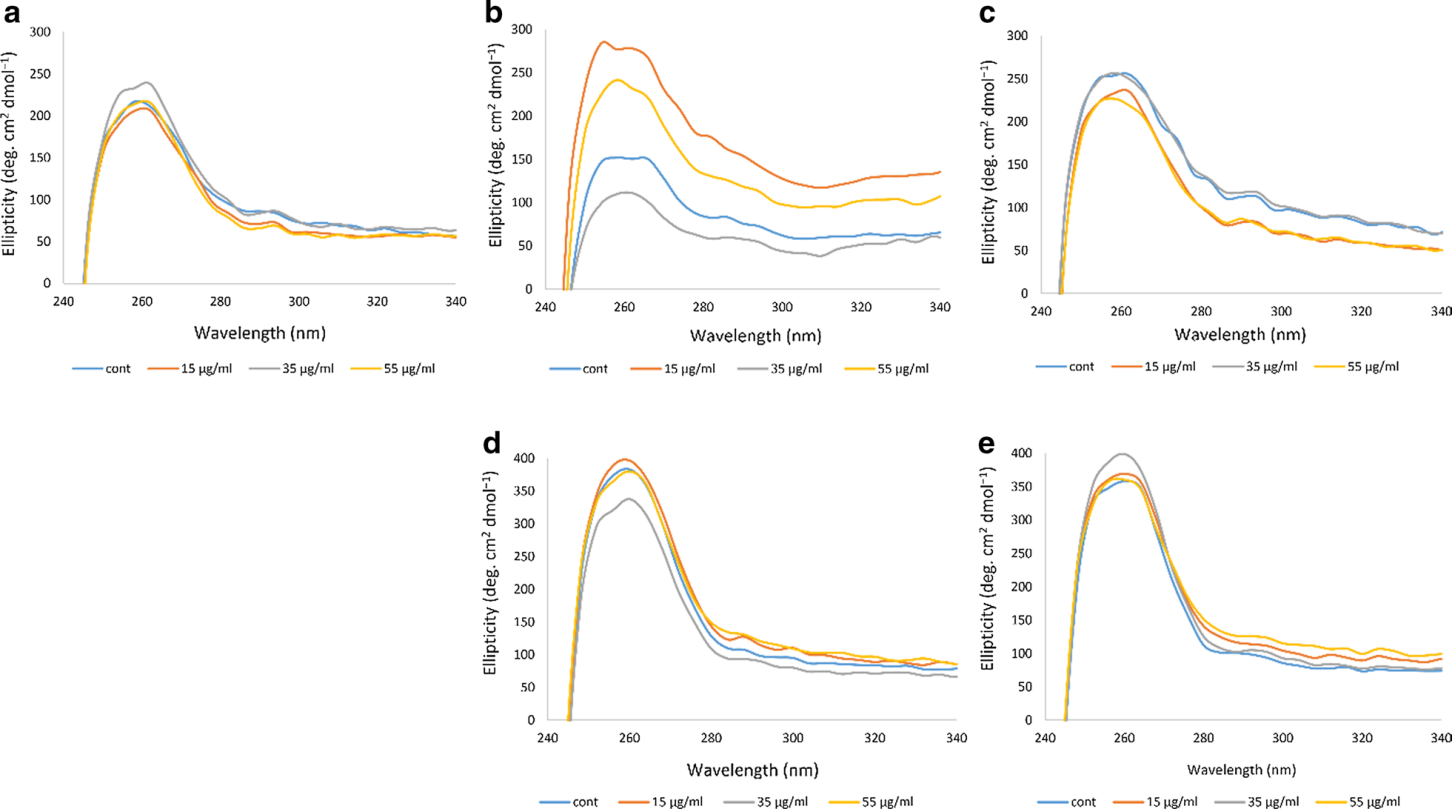
Near-UV CD spectral changes of ozonated whole blood oxy-Hb from five human samples. **a** Near-UV spectra of oxy-Hb for the non-diabetic individual A. **b**–**e** Near-UV spectra of oxy-Hb for the diabetic patients B to E, respectively

Near-UV is also sensitive to changes in the aromatic residues, which reflects the tertiary structure of proteins such as Hb, through a highly positive band at a wavelength of 260 nm [33, 34]. Figure 4 shows the results related to the Hb of the non-diabetic individual and the four diabetic patients. In the Hb samples of the non-diabetic individual A, the different concentrations of ozone had no major effect on the near–UV band and only ozonation at 35 μg/ml caused a slight increase in the intensity of the band. In the Hb samples of the diabetic patient B, changes in the Near-UV band was more evident, such that ozonation at 35 μg/ml caused a great reduction in intensity of the band while ozonation at 55 μg/ml and especially 15 μg/ml greatly increased the intensity of the band compared to the non-ozonated Hb sample. In the diabetic patient C, ozone concentration of 35 μg/ml caused the Near-UV band to be close to the control non-ozonated Hb sample, while ozonation at 15 and 55 μg/ml reduced the intensity of the band. As for the diabetic patient D, the band intensity was overall high and only ozonation at 35 μg/ml resulted in reduction in the intensity of the band.

In the diabetic patient E, ozone concentrations of 15 μg/ml and 55 μg/ml did not show any significant difference and appeared to be similar to the control non-ozonated Hb sample from this patient, while ozone concentration of 35 μg/ml showed a relatively noticeable increase in the intensity of the band at 260 nm.

#### Soret-UV CD

**Fig. 5:**
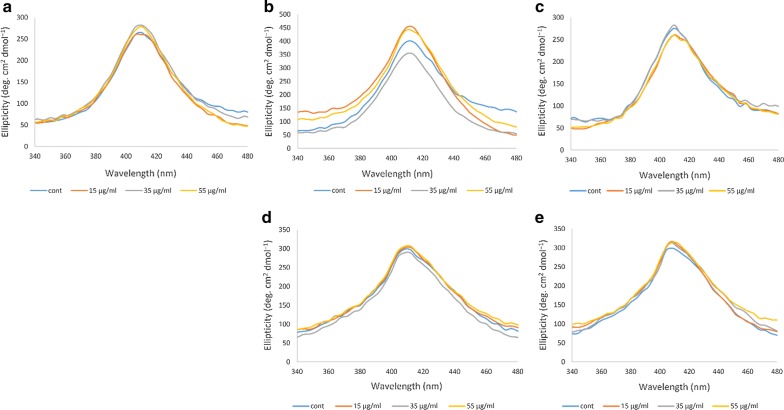
Soret-UV CD spectral changes of ozonated whole blood oxy-Hb from five human samples. **a** Soret-UV spectra of oxy-Hb for the non-diabetic individual A. **b**–**e** Soret-UV spectra of oxy-Hb for the diabetic patients B to E, respectively

The Soret-UV or B-band shows changes in the heme group, which includes binding of the heme group to the globin as well as oxygen. Indeed, when the heme group is attached to the globin, the signal of the Soret region, which is characterized by a highly positive band at 410 nm, is recognizable [31]. Similar to the results seen in the Near-UV studies, Fig. 5 shows that in the Hb samples of the diabetic patient B, changes in the Soret band of Hb exposed to various concentrations of ozone were more pronounced. The intensity of the Soret band, when ozonated with 15 μg/ml and 55 μg/ml ozone increased compared to the non-ozonated Hb sample. However, ozonation at 35 μg/ml, caused a reduction in the Soret band, below the Soret band seen for the non-ozonated sample. As for the non-diabetic individual and the other diabetic patients, the changes were less pronounced.

### UV–VIS absorption spectroscopy

**Fig. 6:**
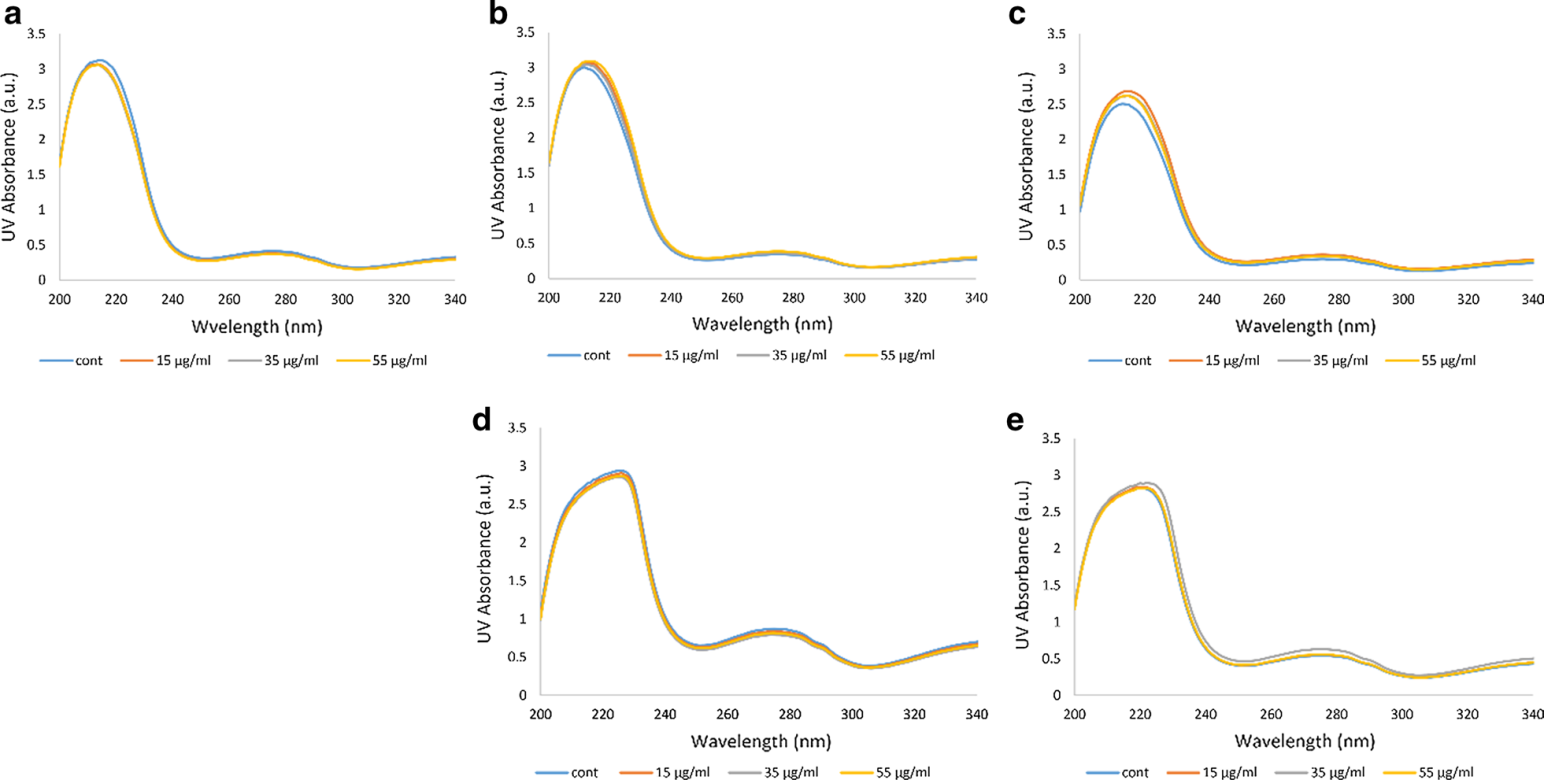
UV–VIS absorption spectral changes related to the peptide and aromatic residue bands. **a** UV–VIS spectra of oxy-Hb for the non-diabetic individual A. **b**–**e** UV–VIS spectra of oxy-Hb for the diabetic patients B to E, respectively. The peaks are related to the 222 nm peptide band and 278 nm aromatic residues band

UV–VIS absorption is caused due to changes in the secondary and tertiary structures of a protein as well as the heme group configuration [31]. UV–VIS absorption of Hb shows peaks representing the presence of the heme prosthetic group recognized by its heterocyclic porphyrin structure [35]. In UV–VIS absorption spectroscopy, peaks at 222 nm and about 278 nm are related to the n → π* transition of amidic bonds and aromatic residues including tryptophan, tyrosine and phenylalanine, respectively [36]. UV–VIS spectra of porphyrin rings are formed from their π electrons detected through 3 original bands: the B-band at 414 nm (same as the Soret band in Soret-UV CD) and a pair of Q-bands at the longer wavelengths in the visible region at 542 and 577 nm [35]. In all Hb samples analysed in this study, the intensity of 222 nm and 278 nm bands remained unchanged, indicating that ozonation did not have a noticeable effect on either the peptide bond or the aromatic residues of the protein (Fig. 6). Having said that, in the diabetic patients D and E, changes occurred in the shape of the peptide band and it shifted towards larger wavelengths (Fig. 6d, e).

**Fig. 7:**
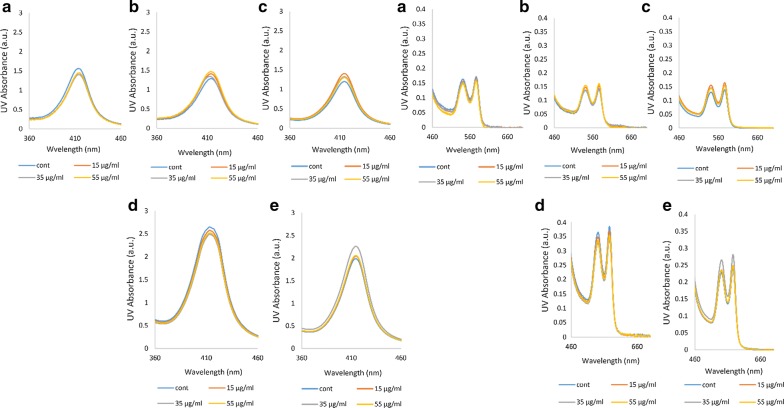
UV–VIS absorption spectral changes related to B- or Soret and Q-bands. **a** UV-VIS spectra of oxy-Hb for the non-diabetic individual A. **b**–**e** UV-VIS spectra of oxy-Hb for the diabetic patients B to E, respectively. Spectral changes related to 414 nm B- or Soret band is shown on the left and that for 542 and 577 nm Q-bands shown on the right hand side of the figure

Overall, looking at the UV–VIS spectra, there are not many significant changes in either the Hb of the non-diabetic individual A or the four diabetic patients in the presence or absence of ozone treatment. However, by closer analysis of the UV–VIS spectra and the Soret and Q-bands in Fig. 7, we find that the diabetic patients D and E, similar to the shape changes seen for their peptide bands and their shift, showed some changes and almost doubling in the band intensities, such that the intensity of the bands increased greatly especially in the Hb samples of patient D (according to data seen for the wavelength range 360–460). Furthermore, there were no signs of methemoglobin formation as a result of ozonation in Hb of either the non-diabetic individual A or the four diabetic patients (according to data seen for the wavelength range 460–660). All Hb samples were in their oxygenated state as identified through the two distinguishable peaks at 542 and 577 nm (Q-bands).

### SDS- and Native-PAGE

**Fig. 8:**
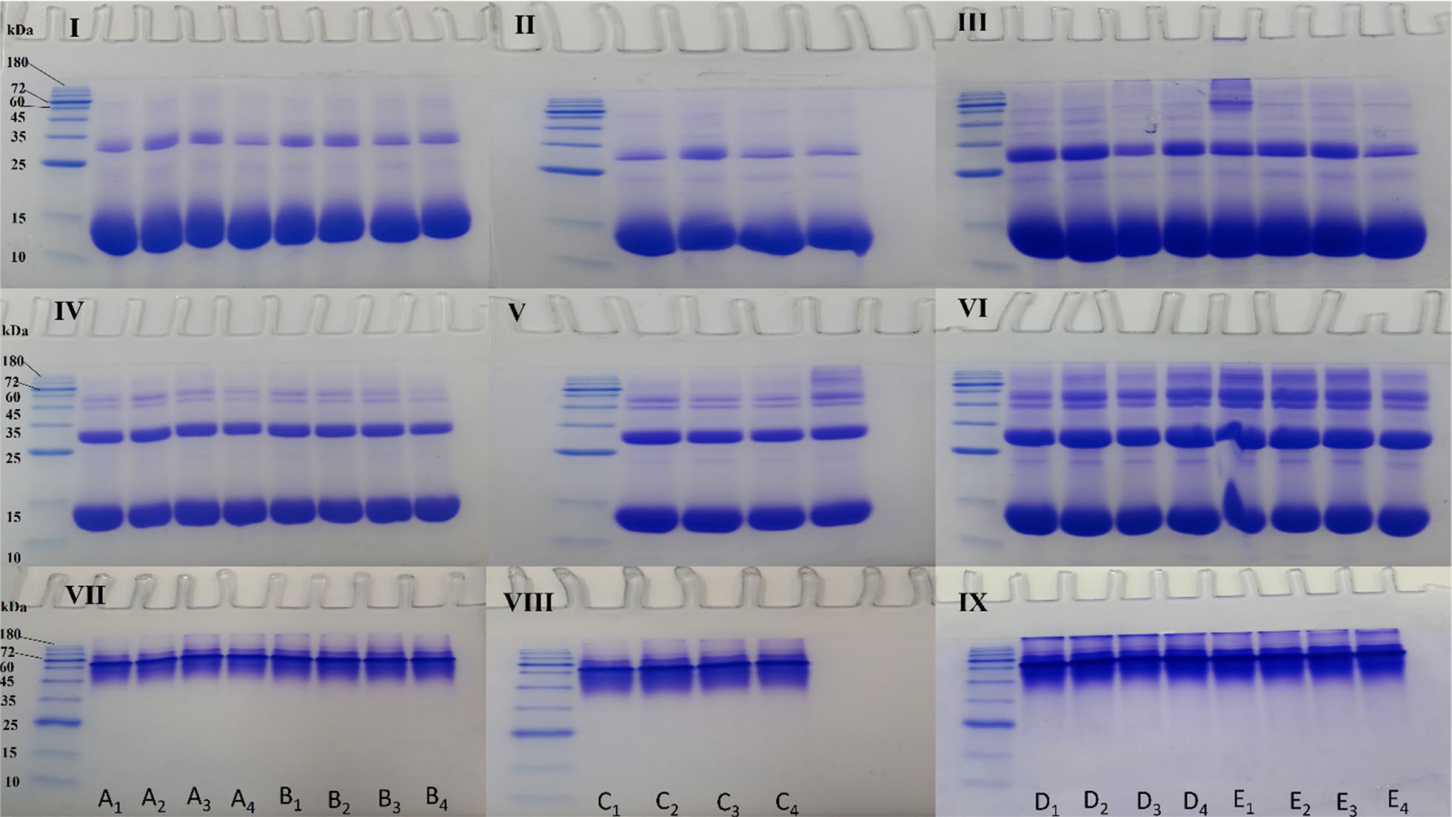
SDS- and Native-PAGE analyses of ozonated whole blood oxy-Hb from five human samples. Gels **I**, **II** and **III** show Hb samples analysed by SDS-PAGE under reducing conditions (in the presence of DTT). Gels **IV**, **V** and **VI**, show Hb samples analysed by SDS-PAGE under non-reducing conditions (in the absence of DTT). Gels **VII**, **VIII** and **IX** show Hb samples analysed by Native-PAGE. Samples A–E include oxy-Hb samples of the non-diabetic individual A and diabetic patients B to E, respectively. Samples in all gels: First lane—Protein marker; Samples numbered 1–4—non-ozonated and ozone treated samples at 15 μg/ml, 35 μg/ml and 55 μg/ml, respectively

Hb is a tetrameric macromolecule composed of four polypeptide chains associated via non-covalent bonds with each polypeptide chain carrying a heme prosthetic group [37]. Protein migration using SDS-PAGE strongly depends on the hydrodynamic properties of the protein, shape and level of the surface charge [38]. In general, we find that there are no significant changes in the Hb monomer and dimer bandwidths in either the non-diabetic individual A or the four diabetic patients and in Hb samples exposed to different concentrations of ozone in both cases, in the presence or absence of DTT as a reducing agent (Fig. 8). However, the major differences are in the appearance of a smear and the formation of higher molecular weight bands such as trimers, tetramers and oligomers. In the diabetic patients B and C and in the non-diabetic individual A, Hb samples (as assessed by SDS-PAGE under both reducing and non-reducing conditions) were found to be less degraded. Furthermore, there were fewer multiple bands or smears seen for Hb of these individuals related to trimers, tetramers and oligomers (Fig. 8, gels I, II, IV and V). However, in the diabetic patients D and E, there was evidence of greater Hb degradation and the presence of multiple higher molecular weight bands and smears, as well as the formation of oligomers showing the instability of Hb in these patients (Fig. 8, gels III and VI). As in our previous work [26], looking at the SDS-PAGE results under reducing conditions (using DTT), we can see a main thick Hb band migrated between the 10 and 15 kDa molecular weight marker bands, indicative of the reduced and denatured alpha and beta globins of Hb with almost similar molecular weights. The next obvious protein band with a lower intensity belongs to a band between the 25 and 35 kDa molecular weight marker bands, indicative of the dimer of Hb. In the presence of DTT, there are additional bands below the 25 kDa marker band and above the 35 kDa marker band, which may be either due to the degradation of Hb by ROS randomly attacking the carbon methene bonds in the tetrapyrrole rings and creating various pyrrole products that can bind together by covalent bonds, as reported previously [39] or be due to the formation of trimers and tetramers of Hb. These additional bands are hardly seen in Hb samples of patients B and C but more evident in Hb samples of patients D and E and especially in the non-ozonated Hb sample of patient E (Fig. 8, gel III). In the absence of DTT, however, the bandwidth of the monomeric Hb band is reduced and added to the dimer bandwidth, which is due to the presence of disulfide linkages, becoming more apparent under non-reducing conditions. In addition, ozonation appeared to have increased the formation of bands indicative of trimers and tetramers and in some cases oligomers through either covalent di-tyrosine cross-links [40] or through disulfide bonds perhaps via the formation of giant extracellular Hb, as previously reported to exist [38], which is more apparent in the Hb samples of patients D and E but also in Hb samples of patient C treated with 55 μg/ml ozone (Fig. 8, gels V and VI). Interestingly, in the case of patient E, ozone at a high concentration of 55 μg/ml had reduced the likelihood of oligomer formation, while this high concentration of ozone resulted in the formation of oligomers in patients C and D (Fig. 8, gels V and VI). In the Hb samples of the non-diabetic individual A, there was no significant effect caused by exposing Hb to different ozone concentrations, such that even at 55 μg/ml ozone, a similar state of Hb was seen compared to the non-ozonated Hb sample. In Hb samples of the diabetic patient B, ozone at different concentrations had little effect on the likelihood of the formation of oligomers and 55 μg/ml of ozone showed similar or better effects than the control non-ozonated Hb sample, in preventing oligomer formation.

In Hb samples of the diabetic patient C and in the presence of DTT, a pronounced reduction in the intensity of the monomer band and increase in the dimer bandwidth was seen when ozonated at 15 μg/ml. Furthermore, in the absence of DTT, ozonation at 55 μg/ml tremendously increased the possibility of oligomer formation. In Hb samples of the diabetic patient D, ozonation at 35 μg/ml showed to reduce the formation of smears and oligomers compared to the other two ozone concentrations of 15 μg/ml and 55 μg/ml. In Hb samples of the diabetic patient E, the non-ozonated Hb control sample revealed to have intense bands indicative of tetramer and oligomer formation. Interestingly, ozonation at 55 μg/ml, decreased the presence of these bands and hence helped to reduce the formation of these oligomeric species. Native-PAGE for Hb of the non-diabetic individual and the four diabetic patients showed the tetramer of Hb as the predominant band near the 60 kDa molecular weight marker band (Fig. 8, gels VII, VIII and IX). Native-PAGE results, in line with the SDS-PAGE results, revealed that the Hb samples of the diabetic patients D and E had more pronounced smear formation and also the existence of oligomers or higher molecular weight species, which as mentioned previously, could be due to the degradation caused by ROS randomly attacking the carbon methene bonds in the tetrapyrrole rings [39] and or the formation of covalent di-tyrosine cross-links [40].

### Dynamic light scattering

**Fig. 9:**
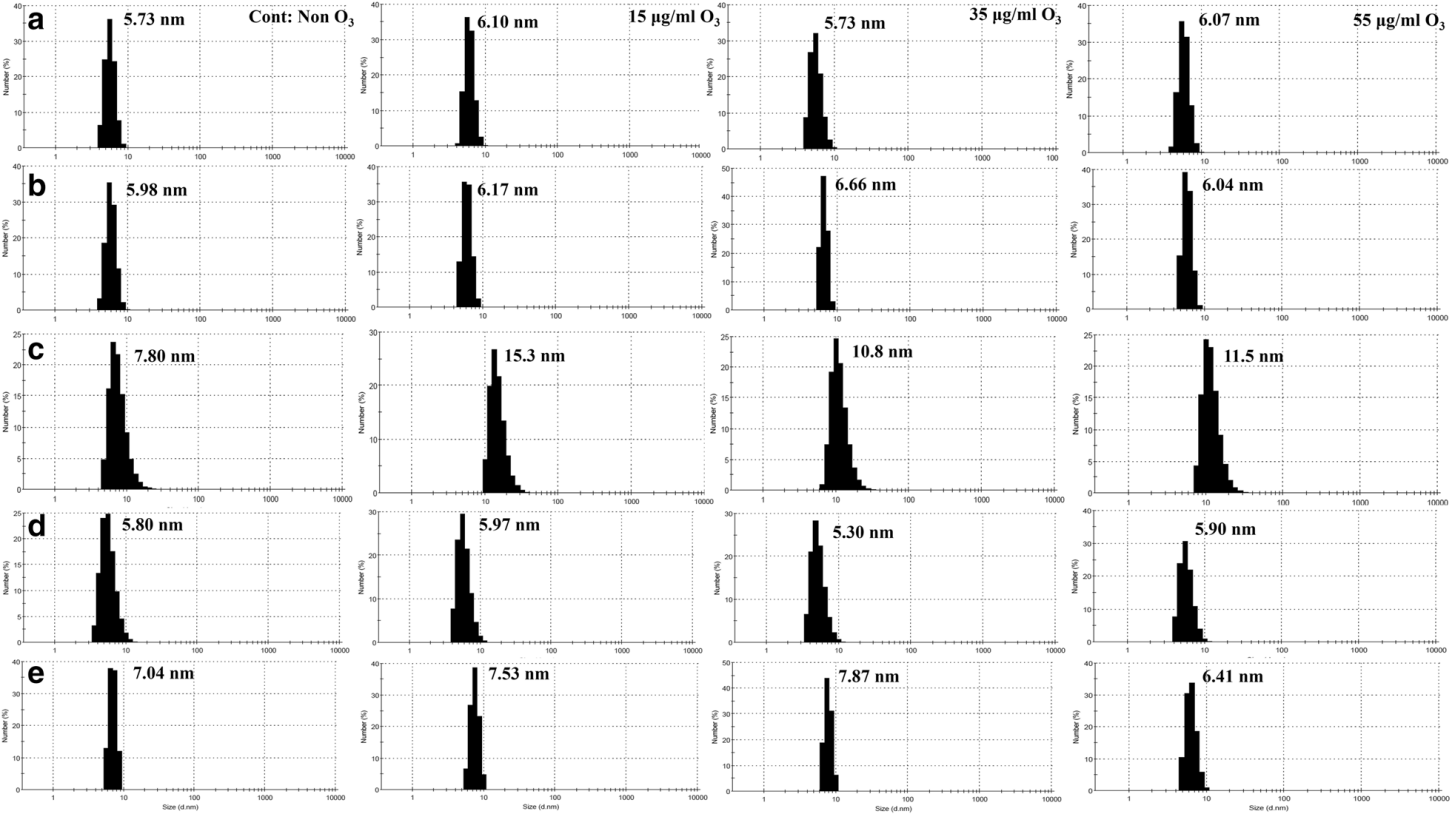
Size distribution revealed by DLS of ozonated whole blood oxy-Hb from five human samples. **a** DLS profiles of oxy-Hb for the non-diabetic individual A. **b**–**e** DLS profiles of oxy-Hb for the diabetic patients B to E, respectively. Column 1—non-ozonated Hb for each individual; Column 2—Hb samples ozonated at 15 μg/ml; Column 3—Hb samples ozonated at 35 μg/ml; and Column 4—Hb samples ozonated at 55 μg/ml. The peak diameter values are given in nanometers. For intensity mode values and percentage intensity please refer to Table 2

DLS results for whole blood oxy-Hb from the non-diabetic individual and the four diabetic patients are given in Table 2 and Fig. 9. In the non-diabetic individual A, ozone concentrations of 15 μg/ml and 55 μg/ml increased the diameter size of the Hb only slightly, while ozonation at 35 μg/ml had no effect on the Hb diameter size in the number mode. In the diabetic patient B, the diameter size of the Hb increased at 35 μg/ml ozone compared to the control non-ozonated Hb sample of the same patient. The Hb diameter for this patient at the other two other concentrations of 15 μg/ml and 55 μg/ml ozone were close to the non-ozonated sample. In the diabetic patient C, all three ozone concentrations increased the Hb diameter size, with the greatest increase in diameter size of Hb detected at 15 μg/ml ozone in the number mode. This is in agreement with the SDS-PAGE results (under reducing conditions) where in gel II of Fig. 8 we observe that at 15 μg/ml of ozone, the probability of Hb degradation and formation of higher molecular weight species increases, and the intensity of the major band for the monomer reduces while the bandwidth of the dimer increases compared to the other Hb samples for this patient. Assessment of the same samples under non-reducing conditions (Fig. 8, gel V), shows that the possibility of forming oligomers at 55 μg/ml of ozone is more pronounced, which is supported by the DLS intensity mode values for this sample (refer to Table 2). In the diabetic patient D, the diameter size of the Hb was reduced when ozonated at 35 μg/ml and instead increased when ozonated at 15 μg/ml and 55 μg/ml. These results are in agreement with the SDS-PAGE results indicating that the lowest probability of oligomer formation is related to the sample ozonated at 35 μg/ml for patient D (Fig. 8, gels III and VI). Furthermore, in the diabetic patient E, the major reduction in Hb diameter size was observable at 55 μg/ml ozone, while the diameter size of Hb ozonated at 15 μg/ml and 35 μg/ml ozone had increased compared to the control non-ozonated Hb sample. In Hb samples for this diabetic patient, ozonation at 55 μg/ml had reduced the formation of oligomers in line with SDS-PAGE results (Fig. 8, gel III and VI) and appeared most significant.

**Table 2:** DLS diameter values in number and intensity modes as well as percentage intensity of ozonated whole blood oxy-Hb of the non-diabetic individual and four diabetic patients

Samples	Ozone concentrations	Diameter (nm) (number mode)	Diameter (nm) (intensity mode)	Intensity % (intensity mode)
Oxy-Hb Non-diabetic individual A	Non-ozonated	5.73	6.23 155 567	18.1 62.7 16
	15 μg/ml	6.10	6.60 112 365	58.3 13.8 27.9
	35 μg/ml	5.73	6.71 166 956	59.6 25.5 13.4
	55 μg/ml	6.07	6.60 129 469	64.1 18.2 17.6
Oxy-Hb Diabetic patient B	Non-ozonated	5.98	6.54 62.5 262	55.2 3 41.8
	15 μg/ml	6.17	6.54 183	41.4 58.6
	35 μg/ml	6.66	6.75 74.5 480	30.4 6.4 63.2
	55 μg/ml	6.04	6.34 168	56.1 43.9
Oxy-Hb Diabetic patient C	Non-ozonated	7.80	26.1 262 4260	85.4 12.3 2.4
	15 μg/ml	15.3	31 271 4470	68.1 24.6 7.3
	35 μg/ml	10.8	25.9 791 3610	88.5 6.5 5
	55 μg/ml	11.5	25 459 4270	89.4 5.7 4.9
Oxy-Hb Diabetic patient D	Non-ozonated	5.8	8.83 317 5100	52.2 29 18.9
	15 μg/ml	5.97	7.87 201 3180	40.7 17.3 41.9
	35 μg/ml	5.30	7.34 289 4740	30.2 25.6 44.1
	55 μg/ml	5.90	7.27 172 1340 Fourth peak not calculated	32.2 14.3 41.4 Fourth peak not calculated
Oxy-Hb Diabetic patient E	Non-ozonated	7.04	7.22 253	44.0 56.0
	15 μg/ml	7.53	7.93 234	48.5 51.5
	35 μg/ml	7.87	8.06 219	45.5 54.5
	55 μg/ml	6.41	7.11 72.3 341	56.4 5.5 38.2

## Discussions

Results from our study place major emphasis on personalizing ozone concentration for any individual wanting to undergo autohemotherapy based on a number of analytical techniques. Although the Hb of the diabetic patients did not show major signs of Hb degradation as depicted by abnormal reduction in Hb colour or methemoglobin formation, explained by the presence of antioxidants and the method of ozone mixing with whole blood in a syringe, but detailed analysis using a number of techniques and in particular SDS-PAGE and DLS, revealed formation of higher molecular weight species or oligomers of Hb. Our findings showed that ozone is able to affect the Hb concentration based on absorbance reading at 280 nm by possibly altering its structure and exposure of aromatic residues. Fluorescence analysis showed that different concentrations of ozone cause changes and polarization in the surrounding environment of the fluorophores (predominantly tryptophans) in the Hb samples. CD analyses including Far-UV (used to analyze the alpha helix peak intensities for Hb samples), Near-UV (used to detect changes in the aromatic residues) and Soret-UV (used to show changes in the heme group in Hb samples) were all used to detect secondary and tertiary structural changes of Hb upon ozonation. Additionally, UV–VIS analysis revealed that no methemoglobin was formed as a result of ozone treatment and all Hb samples remained in the oxy-state. These techniques were all quite informative, however, SDS-PAGE and DLS highlighted major effects of ozone on Hb and appeared to be efficient in providing the necessary information for identifying the recommended safe and effective ozone concentration(s) in O_3_-AHT, as these techniques clearly showed whether or not higher molecular weight species or oligomers are formed. Both these techniques showed the size and state of Hb in terms of oligomerization.

With regards to SDS-PAGE, in the presence of DTT, the probability of Hb degradation and the formation of various pyrrole products and trimer via covalent bonds [39], oligomer formation through covalent di-tyrosine cross-links [40] and in the absence of DTT, the formation of disulfide bonds and perhaps the formation of giant extracellular Hb are observed [38].

In the non-diabetic individual A, analyzed by the three different gels (including reducing and non-reducing SDS-PAGE and Native-PAGE), there was no indication for the formation of oligomers and the samples appeared almost the same as that of the control non-ozonated Hb sample. DLS results of the non-diabetic individual A, also showed that the diameter size of ozonated Hb samples did not change significantly compared to the control. In the diabetic patient B, the probability of forming oligomers was lowest, in the presence of DTT, at the concentration of 15 μg/ml ozone and in the absence of DTT, at 55 μg/ml ozone. DLS results also confirmed this, as the diameter size for Hb ozonated at 55 and 15 μg/ml ozone were 6.04 and 6.17 nm, respectively, only slightly larger than the diameter size of the non-ozonated Hb at 5.98 nm, but significantly smaller than the diameter size for Hb ozonated at 35 μg/ml (6.66 nm).

As for patient C, none of the ozone concentrations seemed appropriate. This is because even though the diameter size for Hb ozonated at 35 μg/ml ozone for this patient was 10.8 nm, significantly smaller than that measured for the other two ozone concentrations (at 11.5 nm and 15.3 nm for 55 μg/ml and 15 μg/ml, respectively), the diameter size of the non-ozonated Hb control sample was much smaller at 7.80 nm. Additionally, SDS-PAGE revealed major oligomerisation at 55 μg/ml ozone for patient C. In patient D, in either the presence or absence of DTT, the concentration of 35 μg/ml ozone reduced the possibility of forming the undesirable oligomeric Hb and absorbance at 280 nm was lower at this ozone concentration compared to 55 μg/ml ozone. DLS also confirmed this as the diameter size for Hb ozonated at 35 μg/ml ozone was 5.30 nm, smaller than the diameter size of the non-ozonated Hb at 5.80 nm. In patient E, in gels with DTT and without DTT and measurement at 280 nm wavelength, the concentration of 55 μg/ml ozone was clearly the most appropriate concentration as it caused the greatest reduction in absorbance reading at 280 nm and reduced the already existing oligomers in the non-ozonated Hb sample of this patient. DLS results also supported this as the diameter size for Hb ozonated at 55 μg/ml ozone was 6.41 nm, significantly smaller than the diameter size of the non-ozonated Hb at 7.04 nm.

## Conclusions

Based on the results obtained from the techniques used in this study, in particular SDS-PAGE and DLS, it can be concluded that in the non-diabetic individual A, there was no significant difference in the Hb samples when using different concentrations of ozone and that it would be safe to use all ozone concentrations, even 55 μg/ml. For the diabetic patient B, it is better to use either 15 μg/ml or 55 μg/ml ozone. For the diabetic patient C, none of the ozone concentrations is recommended. This is because the overall results and in particular DLS results showed that the diameter size of the ozonated Hb had increased compared to the non-ozonated Hb. Ozonation is absolutely hazardous for this patient since it caused the formation of oligomeric species. On the other hand, for the diabetic patient D, it is advisable to use 35 μg/ml ozone. For the diabetic patient E, ozonation at 55 μg/ml is highly recommended as it caused a great reduction in the presence of oligomers, which existed prior to ozonation i.e. in the non-ozonated Hb sample from this patient.

In conclusion, it can be suggested that SDS-PAGE and DLS should be used in order to determine the personalized ozone concentration(s) for a safe and effective autohemotherapy based on blood Hb analysis. The ozone concentration should be highly personalized for each individual undergoing autohemotherapy and should not be generalized in any way.

## Data Availability

Data and material (where applicable) will be available upon request.

## Abbreviations

O_3_-AHT: autohemotherapy
CD: circular dichroism
DTT: dithiothreitol
DLS: dynamic light scattering
Hb: hemoglobin
ROS: reactive oxygen species
TAC: total antioxidant capacity
